# Can radical parametrectomy be omitted in occult cervical cancer after extrafascial hysterectomy?

**DOI:** 10.1186/s40880-015-0041-7

**Published:** 2015-08-08

**Authors:** Huai-Wu Lu, Jing Li, Yun-Yun Liu, Chang-Hao Liu, Guo-Cai Xu, Ling-Ling Xie, Miao-Fang Wu, Zhong-Qiu Lin

**Affiliations:** Department of Gynecologic Oncology, Sun Yat-sen Memorial Hospital, Sun Yat-sen University, No. 107, Yanjiang Road West, Guangzhou, Guangdong 510120 P.R. China

**Keywords:** Radical parametrectomy, Occult cervical cancer, Parametrial involvement, Perioperative complications

## Abstract

**Background:**

Occult invasive cervical cancer discovered after simple hysterectomy is not common, radical parametrectomy (RP) is a preferred option for young women. However, the morbidity of RP was high. The aim of our study is to assess the incidence of parametrial involvement in patients who underwent radical parametrectomy for occult cervical cancer or radical hysterectomy for early-stage cervical cancer and to suggest an algorithm for the triage of patients with occult cervical cancer to avoid RP.

**Methods:**

A total of 13 patients with occult cervical cancer who had undergone RP with an upper vaginectomy and pelvic lymphadenectomy were included in this retrospective study. Data on the clinicopathologic characteristics of the cases were collected. The published literature was also reviewed, and low risk factors for parametrial involvement in early-stage cervical cancer were analyzed.

**Results:**

Of the 13 patients, 9 had a stage IB1 lesion, and 4 had a stage IA2 lesion. There were four patients with grade 1 disease, seven with grade 2 disease, and two with grade 3 disease. The median age of the entire patients was 41 years. The most common indication for extrafascial hysterectomy was cervical intraepithelial neoplasia 3. Three patients had visible lesions measuring 10–30 mm, in diameter and ten patients had cervical stromal invasions with depths ranging from 4 to 9 mm; only one patient had more than 50% stromal invasion, and four patients had lymph-vascular space invasion (LVSI). Perioperative complications included intraoperative bowel injury, blood transfusion, vesico-vaginal fistula, and ileus (1 case for each). Postoperative pathologic examination results did not show residual disease or parametrial involvement. One patient with positive lymph nodes received concurrent radiation therapy. Only one patient experienced recurrence.

**Conclusions:**

Perioperative complications following RP were common, whereas the incidence of parametrial involvement was very low among selected early-stage cervical cancer patients. Based on these results, we thought that patients with very low-risk parametrial involvement(tumor size ≤2 cm, no LVSI, less than 50% stromal invasion, negative lymph nodes) may benefit from omitting RP. Further prospective data are warranted.

## Background

Occult invasive cervical cancer discovered after extrafascial hysterectomy is rare. Without any further therapy, the 5-year survival rate of patients with occult invasive cervical cancer is less than 50% [1]. The treatment of occult invasive cervical cancer includes (1) adjuvant radiation therapy [2, 3] or (2) radical parametrectomy which usually performed with upper vaginectomy and pelvic lymphadenectomy [4–6]. However, no prospective randomized controlled trials have compared the efficacy of these two treatments due to the limited number of cases. Radiation therapy frequently results in bladder, rectal, and sexual dysfunction and, particularly, loss of ovarian function. In young women, RP is usually recommended, because a negative pathology finding indicates no need for further therapy [7]. At the same time, the incidence of operative complications increase with the performance of radical parametrectomy, with reported rates as high as 30%. The complications include intraoperative blood loss, blood transfusion, incidental cystostomy, and postoperative ileus [8]. In cervical cancer surgery, individualization of treatment to reduce therapy-associated morbidity is the most current trend. Several retrospective studies in recent years have shown that the incidence of parametrial involvement is very low in selected patients [9–15]; therefore, it is likely unnecessary to perform parametrectomy in these patients. The purpose of this study was to identify a subgroup of patients at low risk for parametrial involvement who may not require RP for occult cervical cancer.

## Patients and methods

Thirteen patients who underwent radical parametrectomy (RP) with upper vaginectomy and pelvic lymphadenectomy in the Department of Gynecology of Sun Yat-sen Memorial Hospital between July 2013 and Jan 2005 were included in this study. All of the patients had been referred to our department after prior total hysterectomies at other hospitals. Prior to undergoing RP, informed consent was obtained from all patients.

The patients who met the following criteria were considered eligible for RP: (1) a normal pelvic examination before surgery and (2) no evidence of residual lesions on the vaginal cuff. Furthermore, samples from the extrafascial hysterectomy were pathologically reviewed to exclude patients with micro-invasive lesions (IA_1_) or patients with cancer involving the hysterectomy margin. Preoperative evaluations with magnetic resonance imaging (MRI) or computed tomography (CT) were performed to exclude lymph node involvement.

Clinicopathologic data were collected, including demographics, indications for first surgery, tumor stage, review of the prior surgical specimen’s pathology, presence of lymph-vascular space invasion (LVSI), nodal status, operative complications, duration of indwelling catheterization, residual urine volume, duration of hospital stay, recurrence, and survival. Follow-up data were obtained by medical record review or by patient correspondence.

## Results

### Clinicopathologic characteristics of patients at the initial operation

The median age of the 13 patients was 41 years (range 35–58 years). The median follow-up was 61 months (range 15–109 months). The patients’ clinical characteristics associated with the prior surgery are shown in Table 1.

**Table 1:** Clinicopathologic characteristics of patients with early-stage cervical cancer at the initial operation

No.	Age (years)	Previous diagnosis	Previous surgery	Initial surgery pathologic characteristics						FIGO stage
				Histology	Depth of stromal invasion	LVSI	Depth of invasion (mm)	Tumor size (mm)	Grade	
1	47	CIN3	TAH-LSO	SCC	<1/2	−	/	12	G2	IB1
2	42	CIN3	TAH	SCC	<1/2	−	5	/	G1	IA2
3	40	AUB	TAH	SCC	<1/2	−	8	/	G2	IB1
4	35	CIN3	TAH	SCC	<1/2	+	7	/	G1	IB1
5	55	Uterine prolapse	TVH	CAC	<1/2	+	4	/	G3	IA2
6	39	Myoma	TAH	SCC	<1/2	−	7	/	G2	IB1
7	41	AUB	TAH	SCC	<1/2	+	9	/	G2	IB1
8	37	CIN3	TAH	SCC	>1/2	−	/	30	G2	IB1
9	38	Adenomyosis	TAH	CAC	<1/2	−	6	/	G2	IB1
10	37	CIN3	TAH	SCC	<1/2	+	8	/	G3	IB1
11	44	AUB	TAH	SCC	<1/2	−	5	/	G1	IA2
12	58	Uterine Prolapse	TVH	CAC	<1/2	−	5	/	G2	IA2
13	41	Myoma	TAH	SCC	<1/2	−	/	10	G1	IB1

*CIN* cervical intraepithelial neoplasia, *AUB* abnormal uterine bleeding, *TAH* total abdominal hysterectomy, *LSO* left salpingo-oophorectomy, *TVH* total vaginal hysterectomy, *SCC* squamous cervical cancer, *CAC* cervical adenocarcinoma, *LVSI* lymph-vascular space invasion. “+” positive, “−” negative.

The indications for the initial surgery were CIN3 (*n* = 5), abnormal uterine bleeding (AUB) (*n* = 3), uterine prolapse (*n* = 2), myoma (*n* = 2), and adenomyosis (*n* = 1). The previous hysterectomy had been performed vaginally (*n* = 2) or by laparotomy (*n* = 11).

Nine patients were diagnosed with squamous carcinoma, and four patients had adenocarcinoma. There were nine patients with stage IB1 disease and 4fourwith stage IA2 disease. Only one patient had deep stromal invasion. Three patients had visible lesions measuring 10–30 mm, and ten had no visible lesions but invasions with depths ranging from 4 to 9 mm. Only four patients had LVSI. Four patients had grade 1 disease, seven had grade 2 disease, and two had grade 3 disease.

### Clinicopathologic characteristics of patients treated with RP

The clinicopathologic characteristics of the patients who underwent RP are listed in Table 2. Parametrectomy was performed a median of 20 days after the prior surgery (range 12–30 days). The mean duration of surgery was 242 min (range 200–270 min). The estimated average blood loss was 400 mL (range 300–500 mL).

**Table 2:** Clinicopathologic characteristics of patients with early-stage cervical cancer receiving radical parametrectomy

No.	Interval time (days)	Operative time (min)	Blood loss (mL)	Transfusion (mL)	Postoperative pathology			Intraoperative complication	Postoperative complications	Duration of indwelling catheter (days)	First-time urine residual volume (mL)	Duration of hospital stay (days)	Adjuvant therapy
					Residual	LN	Para						
1	18	270	300	−	−	−	−	Intestinal injury	−	14	100	19	−
2	14	245	450	−	−	−	−	−	−	20	200	20	−
3	12	265	400	−	−	−	−	−	−	16	130	16	−
4	20	250	420	−	−	−	−	−	Vesicovaginal fistula	14	80	14	Repair surgery
5	21	235	400	−	−	−	−	−	Ileus	20	240	30	−
6	30	250	350	−	−	−	−	−	−	14	90	16	−
7	16	270	500	400	−	−	−	−	−	14	80	18	−
8	25	250	400	−	−	+	−	−	−	15	150	16	Chemoradiotherapy
9	28	240	500	−	−	−	−	−	−	18	100	18	−
10	19	200	300	−	−	−	−	−	−	14	50	18	−
11	20	220	350	−	−	−	−	−	−	14	60	14	−
12	30	235	400	−	−	−	−	−	−	14	100	14	−
13	15	210	400	−	−	−	−	−	−	30	250	15	−

*LN* lymph node, *Para* parametrium. “+” positive, “−” negative.

There were two intraoperative complications. One patient had an intestinal injury due to pelvic adhesions from a previous surgery. An ileal anastomosis was subsequently performed, and the patient recovered well. The other patient received a blood transfusion due to preoperative anemia and severe intraoperative blood loss.

Two postoperative complications occurred. One patient had copious vaginal discharge after the catheter was removed. A methylene blue test was performed, and the diagnosis of a vesicovaginal fistula was confirmed. A repair surgery was successfully conducted 3 months after the diagnosis. The other patient had a postoperative ileus 6 days after the operation and recovered after conservative treatment.

The catheter was removed a median of 14 days (range 14–30 days) after RP, and the median residual urine volume was 100 mL (range 50–250 mL). The median hospital stay was 16 days (range 14–30 days).

All patients who underwent RP had no residual disease found in their radical parametrectomy specimen, and only one patient had nodal metastasis. Specimens from the previous surgery showed that the tumor size was 30 mm in diameter and revealed deep stromal invasion. The patient received concurrent chemoradiation therapy (CCRT) and showed no evidence of recurrence during follow-up.

Only one patient (No. 10) experienced recurrence on the vaginal cuff during a median follow-up of 61 months (range 15–109 months). The initial pathologic diagnosis of this patient was poorly differentiated squamous cell carcinoma with positive LVSI, which was consistent with the diagnosis of the review. The patient was treated with whole pelvic radiation and brachytherapy. No further evidence of recurrence was detected during follow-up.

## Discussion

Although occult cervical cancer discovered after extrafascial hysterectomy for benign indications or pre-invasive cervical cancer is uncommon, the management of this cancer is debated. Careful evaluation to exclude cervical malignancy is necessary before performing a simple hysterectomy. The evaluation of a benign lesion should include a “3-step” screening procedure that includes cervical cytology, colposcopy, and biopsy, whereas loop excision or conization is strongly recommended for pre-invasive cervical lesions.

However, due to false negatives, a few patients who undergo simple hysterectomy may have occult cervical cancer even after appropriate preoperative cervical evaluation. Extrafascial hysterectomy is not sufficient for patients with disease beyond stage IA1 because the survival rate is less than 50%, whereas the survival rate of patients who receive radical surgery or radiation therapy is up to 89% [1]. The options for treatment of patients with recurrence include: (1) a second surgery with RP, upper vaginectomy and pelvic lymphadenectomy, or (2) adjuvant radiation. However, due to the limited number of cases, no prospective randomized controlled trial has compared the efficacy of these two methods. Park et al. [16] retrospectively evaluated the outcomes in 99 patients with occult cervical cancer who had IA2-IIA lesions. Of the 99 patients, 26 received no definitive treatment, 44 received radiation therapy (RT) or CCRT, and 29 underwent RP. After a median follow-up of 116 months, the recurrence rates were 34.6%, 6.8%, and 0% in the observation, RT, and RP groups, respectively. Although RP and RT/CCRT yielded similar therapeutic efficacy, late complications related to RT were more common and more difficult to address than the perioperative complications related to RP.

Radical parametrectomy was first described by Daniel and Brunschwig [4]. The procedure is usually performed with an upper vaginectomy and removal of the pelvic and/or para-aortic lymph nodes. However, the incidence of incidental injury may arise due to destruction of the normal structure and pelvic adhesions caused by the previous surgery. Perioperative complications related to RP were reported in approximately 30% of patients [8]. Intraoperative blood transfusion, incidental cystostomy, and postoperative ileus were the most common morbidities [7, 8]. Kinney et al. [6] reported 27 patients who underwent RP. In their study, 24 patients (89%) needed an intraoperative blood transfusion and 2 patients (7%) were diagnosed with a vesicovaginal fistula. Leath et al. [8] reported that 30% of 23 patients had operative complications as follows: blood transfusion (17%), incidental cystostomy (9%), and postoperative ileus (4%). There were four complications in our study, including one blood transfusion, one incidental bowel injury, one vesicovaginal fistula, and one postoperative ileus, which is consistent with the results of literature [7, 8]. RP may simultaneously remove the residual ligaments and autonomic nerves, which may result in urination dysfunction. In our study, 46.2% of the catheters were removed more than 14 days after surgery. Adhesions from the previous surgery, which increase the risk of nerve injury, were likely the main cause of longer preserve time of catheter.

Most surgeons are unwilling to perform RP because they consider the RP procedure to be difficult and be associated with a high rate of perioperative complications. Moreover, some surgeons even question the necessity of parametrial resection. In their opinions, not all patients with cervical cancer need to undergo a radical parametrial resection because the incidence of parametrial involvement is not high in early-stage cervical cancer, with a rate of 0.0%–7.7% in stage IA2–IB1 disease [13] and 8.4%–10.4% in stage IB1 disease [12, 14, 17]. In recent years, many retrospective studies have reported that the incidence of parametrial involvement is very low in select patients [9–15]. The authors discussed how to identify these patients and whether they could be considered candidates for less radical surgery such as modified radical hysterectomy or extrafascial hysterectomy [8, 15, 18, 19]. Kinney et al. [9] studied 387 patients with stage IB1 cervical cancer and found that the risk of parametrial involvement was 0% in patients with a tumor size smaller than 2 cm and no LVSI. Stegeman et al. [11] reported on early-stage cervical cancer with a tumor size <2 cm, infiltration depth <10 mm, negative pelvic lymph nodes, and absent LVSI and found the risk of parametrial involvement to be 0.63%. Gemer et al. [15] found that in patients with negative LVSI, tumor size ≤2 cm, and negative lymph nodes, the risk of parametrial involvement was 0.0%. Kodama et al. [14] reviewed 200 stage IB1 cervical cancer cases and concluded that in patients with LVSI, an infiltration depth <10 mm, and age ≤50 years, the risk of parametrial involvement was 0.0%. Wright et al. [12] reported an incidence of parametrial involvement in patients with cervical cancer of 10.8%, but in patients with a tumor size <2 cm, absent LVSI, and negative lymph nodes, the parametrial involvement rate was 0.4%. Covens et al. [10] studied 842 patients with early-stage cervical cancer and found that in patients with an infiltration depth <10 mm, tumor size ≤2 cm, and negative lymph nodes, the risk of parametrial involvement was 0.6%. Frumovitz et al. [13] studied the incidence of parametrial involvement in 125 patients with stage IA2–IB1 cervical cancer and found that in patients with tumor size ≤2 cm and absent LVSI, the incidence was 0.0%. The relationship between the parametrial involvement rate and favorable pathologic characteristics is listed in Table 3. In our study, there were four LVSI-positive patients, one patient with lymph node involvement, and one patient with a tumor size >2 cm; however, interestingly, there was no parametrial involvement noted, which may be a result of the limited number of cases. Only one patient experienced recurrence on the vaginal cuff. The pathology was conformed as poorly differentiated squamous cell carcinoma with positive LVSI.

**Table 3:** Parametrial involvement rates in patients with early-stage cervical cancer with favorable pathologic characteristics

References	No.	Stage	Low risk criteria					Parametrial involvement (%)
			Tumor size	Depth of invasion	LVSI	LN involvement	Age (years)	
Gemer et al. [15]	530	IA2–IB1	≤2 cm	N	−	−	N	0
Kodama et al. [14]	200	IB1	N	<10 mm	−	N	<50	0
Frumovitz et al. [13]	125	IA2–IB1	≤2 cm	N	−	N	N	0
Stegeman et al. [11]	103	IA2–IB1	<2 cm	<10 mm	−	−	N	0.63
Wright et al. [12]	594	IA–IIA	<2 cm	N	−	−	N	0.4
Covens et al. [10]	842	IA–IB1	≤2 cm	≤10 mm	−	−	N	0.6
Kinney et al. [9]	387	IB1	<2 cm	N	−	N	N	0

*LVSI* lymphovascular space involvement, *LN* lymph node, *N* not mentioned. “+” positive, “–” negative.

Because the incidence of parametrial involvement was very low in selected early-stage cervical cancer cases and the incidence of complications caused by RP were high, the portion of patients who would have benefited from RP was low. Triaging patients with a low risk of parametrial involvement to omit RP is theoretically feasible. Parametrial involvement is associated with tumor size, LVSI, and infiltration depth, which may be obtained by reevaluating the pathologic specimens from the initial surgery. The pelvic lymph node status can be assessed by imaging such as MRI, CT, or positron emission tomography (PET)-CT before reoperation. A sentinel lymph node (SLN) biopsy can also be performed to evaluate the pelvic lymph node status and parametrial involvement. Strnad et al. [20] studied 158 patients with stage IA2-IB1 cervical cancer and found that in patients with negative SLNs, the incidence of parametrial involvement was 0%, suggesting that SLN-negative patients can avoid unnecessary RP and even systematic pelvic lymph node resection. Pluta et al. [19] conducted a pilot study to evaluate the feasibility and safety of less radical surgery. Sixty patients with stage IA1–IB1 cervical cancer were included in the study. Patients were selected based on favorable cervical tumors (IA1 with LVSI, IA2 and IB1 with tumor size <20 mm, and less than 50% stromal invasion). All patients underwent laparoscopic SLN identification using frozen sections (FS). SLN-negative patients underwent complete pelvic laparoscopic lymphadenectomy and vaginal hysterectomy and FS-positive patients underwent radical hysterectomy with low para-aortic lymphadenectomy. There were no recurrences in 55 SLN-negative patients or in 5 SLN-positive patients during the follow-up (median 47 months; range 12–92 months). This preliminary study showed that it is both feasible and safe to reduce the radicality of parametrial resection in cases of small tumor volume in SLN-negative patients.

The main limitation of our study was an insufficient sample size; furthermore, it was not a randomized controlled trial. Thus, our data cannot be used to justify omitting RP in selected patients with occult cervical cancer after extrafascial hysterectomy.

## Conclusions

In summary, although RP is effective in patients with occult cervical cancer after extrafascial hysterectomy, its surgery-related complications must be considered. Our results suggest that a group of patients with very low-risk parametrial involvement may benefit from omitting RP. Randomized controlled trials are warranted to assess the feasibility and efficacy of extrafascial hysterectomy in patients with low-risk parametrial involvement.

## Abbreviations

RP: radical parametrectomy
CIN3: cervical intraepithelial neoplasia3
CT: computed tomography
RT: radiation therapy
CCRT: concurrent radiation therapy
SLN: sentinel lymph node
AUB: abnormal uterine bleeding
TAH: total abdominal hysterectomy
LSO: left salpingo-oophorectomy
TVH: total vaginal hysterectomy
SCC: squamous cervical cancer
CAC: cervical adenocarcinoma
LVSI: lymph-vascular space invasion
LN: lymph node
Para: parametrium
FS: frozen sections
MRI: magnetic resonance imaging
